# Targeted chondrogenic differentiation of human MSCs using niosomes for SOX9 gene delivery: comparison of minicircle and conventional plasmids

**DOI:** 10.1186/s13287-025-04867-5

**Published:** 2025-12-25

**Authors:** Junquera López-Seijas, Alba Iglesias-Fente, Alba Ramil-Bouzas, Sara Paniagua-Barro, Juan Fafián-Labora, Ana Rey-Rico

**Affiliations:** 1 Centro Interdisciplinar de Química e Bioloxía - CICA, Universidade da Coruña, 15071 A Coruña, Spain; 2 Departamento de Bioloxía, Facultade de Ciencias, Universidade da Coruña, 15071 A Coruña, Spain; 3 Instituto de Investigación Biomédica de A Coruña (INIBIC), Complexo Hospitalario Universitario de A Coruña (CHUAC), Servizo Galego de Saúde (SERGAS), 15006, A Coruña, Spain; 4 Departamento de Fisioterapia, Medicina y Ciencias Biomédicas, Facultad de Ciencias de la Salud, Universidade da Coruña (UDC), Campus de Elviña, 15071 A Coruña, Spain; 5 Grupo de Investigación en Reumatología y salud (GIR-S), Centro Interdisciplinar de Química e Bioloxía - CICA, INIBIC-Sergas, Universidade de A Coruña (UDC), 15071 A Coruña, Spain

**Keywords:** Gene delivery, Niosomes, DP20CQ, DP80CH, hMSCs, Minicircle DNA, Parental plasmid, SOX9, Chondrogenesis

## Abstract

**Background:**

Niosomes represent a promising non-viral gene delivery system, offering an alternative to viral vectors for the genetic modification of hard-to-transfect cells, such as mesenchymal stem cells (MSCs), which are pivotal in regenerative medicine. Specifically, SOX9 gene transfer is a valuable strategy for cartilage tissue repair, as it promotes chondrocyte differentiation while repressing hypertrophic and osteogenic markers. In this study, we investigated the potential of niosomes to deliver SOX9, using both parental and minicircle plasmids, to induce chondrogenic differentiation in primary bone marrow-derived human MSCs (hMSCs).

**Methods:**

Niosomes were synthesised using the thin-film hydration method and complexed with either parental or minicircle SOX9 plasmids to form nioplexes. Physicochemical properties of niosomes and nioplexes were studied in terms of size, zeta potential, complexation, and protection capacity. Primary hMSCs were transfected in a 2D monolayer and 3D aggregate cultures using Lipofectamine as a positive control of transfection. Chondrogenic differentiation was assessed by gene expression (SOX9, ACAN, COLII, COLI, COLX), histological and immunohistochemical staining (Toluidine blue, haematoxylin & eosin and SOX9, COLII, COLI, COLX, respectively), and biochemical (proteoglycans, DNA and protein contents) analyses of main cartilage markers.

**Results:**

SOX9 delivery via DP20CQ niosome systems significantly enhanced the expression of key chondrogenic markers (SOX9, ACAN, and COLII) and increased production of a characteristic hyaline-like cartilage matrix. In contrast, Lipofectamine-based complexes induced hypertrophic and fibrocartilaginous phenotypes, evidenced by increased expression of COLX and COLI. Quantification of proteoglycan production, along with proteins and DNA content, supported these findings. Both plasmid types promoted comparable chondrogenic outcomes, but parental plasmids yielded more consistent results than minicircles.

**Conclusions:**

Delivery of SOX9 plasmids via niosomes promotes enhanced chondrogenic differentiation of primary hMSCs in a 3D aggregate culture system, leading to the formation of hyaline-like cartilage tissue. This non-viral strategy represents a promising gene delivery platform for cartilage reparative therapies.

**Supplementary Information:**

The online version contains supplementary material available at 10.1186/s13287-025-04867-5.

## Background

Medical advances have extended human lifespan, leading to a higher prevalence of age-related degenerative diseases, particularly musculoskeletal disorders. Unlike bone, cartilage has a limited self-repair capacity due to its aneural, avascular, and alymphatic nature. Moreover, current treatments fail to fully restore native hyaline cartilage. Therefore, untreated cartilage injuries can progress to osteoarthritis, a degenerative joint disease affecting over 500 million people worldwide and representing one of the leading causes of chronic disability [1].

Given these challenges, mesenchymal stem cells (MSCs) have emerged as a key cell population for cartilage tissue repair, owing to their ability to differentiate into multiple mesodermal lineages, modulate immune responses, secrete bioactive paracrine factors, and regulate the synthesis of extracellular matrix components. Marrow stimulation techniques have been widely used in clinics for recruiting progenitor cells from the underlying bone marrow to the defect´s site. However, these procedures do not provide a standardised MSC-based therapy [2, 3] and often lead to the formation of fibrocartilage, a tissue rich in type-I collagen rather than the type-II collagen, characteristic of native articular hyaline cartilage [1, 4]. In light of this, several studies have reported reduced proliferation of MSCs within the hostile microenvironment of the injured tissue, impairing nutrient availability and inducing cell death [5]. These factors significantly compromise the capacity of MSCs to secrete soluble factors necessary for effective tissue repair [4, 6, 7].

Gene delivery to MSCs has gained attention as a promising strategy to overcome these limitations by promoting the expression of paracrine factors and reinforcing the reparative properties of this remarkable cell population. Current gene transfer methods for MSCs include viral and non-viral vectors [8]. Viral vectors provide high transfection efficiency but face safety and manufacturing challenges. Alternatively, non-viral vectors, typically cationic lipids or polymers, circumvent many biosafety concerns and offer more versatility, although their efficiency remains lower, particularly in primary cultures such as MSCs [9, 10].

Among non‑viral carriers, niosomes have recently emerged as gene vectors specifically tailored for MSCs transfection, due to their biocompatibility and adaptability [11–14]. Niosomes are vesicular nanocarriers predominantly composed of non-ionic surfactants (polysorbates [15] or Spans [16]), combined with cationic lipids (e.g., 1,2-di-O-octadecenyl-3-trimethylammonium propane, DOTMA [11]) and helper lipids (e.g., cholesterol [17], chloroquine [18] or squalene [13, 19]), enabling efficient complexation of genetic cargo through electrostatic interactions. Compared to conventional liposomes, niosomes exhibit improved chemical stability, cost-effectiveness, extended storage, and reduced immunogenicity [20]. Importantly, recent studies have demonstrated the potential of niosomes to significantly increase gene transfer efficiency in MSCs, a cell type widely considered challenging to transfect [14, 21]. In this study, niosomes composed of DOTMA (D) as the cationic lipid, polysorbate 20 (P20) as the non-ionic surfactant, and chloroquine (CQ) as the helper lipid proved to have suitable physicochemical characteristics and efficient genetic modification of human MSCs (hMSCs). These features underscore the potential of niosomes as safe and versatile non-viral vectors for advancing MSCs-based regenerative therapies [21].

Beyond the delivery vector, the genetic cargo has a major influence on determining the success of gene transfer outcomes. Notably, conventional plasmids often contain bacterial backbone sequences, such as antibiotic resistance genes and origins of replication, which are unnecessary for gene expression in mammalian cells and can trigger immunogenic responses and transcriptional silencing [22]. By contrast, minicircle DNA vectors, lacking these regions, may provide superior and prolonged transgene expression [23–25].

Based on the abovementioned, in the present study, we aimed, for the first time to the best of our knowledge, to design a novel gene delivery system combining niosomes and DNA minicircles to promote SOX9-driven chondrogenesis in bone marrow-derived hMSCs. Two niosome formulations previously optimised in immortalised hMSCs [14] were tested in two-dimensional (2D) hMSC monolayer cultures, and the most efficient combination with parental or minicircle plasmids was subsequently evaluated in a three-dimensional (3D) hMSC aggregate model.

Results from this study highlight the potential of DP20CQ-based niosomes as a reliable and efficient platform for genetically engineering hMSCs, enabling the delivery of therapeutic genes, such as SOX9, to enhance hyaline-like cartilage matrix deposition while limiting hypertrophy, an essential step toward articular cartilage repair.

## Materials and methods

### Materials

Polysorbate 20 (P20, MW 1105 g/mol), polysorbate 80 (P80, MW 1064 g/mol), and chloroquine (CQ, MW 515.5 g/mol) were obtained from Alfa Aesar (USA). 1,2-di-O-octadecenyl-3-trimethylammonium propane (D, DOTMA, MW 670.6 g/mol) was purchased from Angene (China). Dulbecco’s Modified Eagle’s Medium (DMEM), Phosphate-Buffered Saline (PBS), Fetal Bovine Serum (FBS), Penicillin/Streptomycin (P/S), OptiMEM™ reduced serum medium, Insulin-Transferrin-Selenium-A (ITS), dichloromethane, and cholesterol 95% (CH, MW 386.7 g/mol) were sourced from Gibco -Thermo Fisher Scientific (USA). SalI and XbaI restriction enzymes, LB Agar, Tris-Borate-EDTA (TBE) buffer, DNase I (1000 U), agarose, Sodium Dodecyl Sulfate (SDS, MW 288.4 g/mol), Halt™ protease, and phosphatase inhibitor cocktail (100X), and Micro BCA™ Protein Assay Kit were obtained from Thermo Fisher Scientific (USA). Lipofectamine^®^ Stem Reagent (LPF), SYBR Green Gold (10000 X), Luria Broth (LB), 5X annexin-binding buffer, and HiPure Plasmid Maxiprep Kit were purchased from Invitrogen - Thermo Fisher Scientific (USA). The Human SOX9 (Transcription factor SOX-9) ELISA Kit was acquired from FineTest (China). Recombinant TGF-β3 was obtained from Peprotech (USA).

MN511A-SOX9 construct, kanamycin, growth media, and induction media were acquired from System Biosciences (USA). Annexin V APC (1:10), Propidium Iodide staining solution (50 µg/mL; 1:100), PE-conjugated anti-human CD34, FITC-conjugated anti-human CD45, PE-conjugated anti-human CD73, FITC-conjugated anti-human CD90, FITC-conjugated anti-human CD105, FITC, and PE isotypes were obtained from BD Biosciences (USA).

Uranyl acetate, 1,9-Dimethyl-Methylene Blue Dye (DMMB), toluidine blue, sodium L-ascorbate, dexamethasone, and sodium pyruvate were purchased from Sigma-Aldrich (USA). Eosin Y and Harris haematoxylin solution were obtained from Carl Roth (Germany), and Chondroitin Sulfate C Sodium Salt from Toronto Research Chemical (Canada).

The First Strand cDNA Synthesis Kit for RT-PCR was obtained from Roche (Switzerland), RNeasy Protect Mini Kit from Qiagen (Germany), and PowerUp™ SYBR™ Green Master Mix from Applied Biosystems (USA).

The anti-type II collagen (II-II6B3, COLII) antibody was purchased from DSHB (USA) and the anti-type X collagen (COLX) from Sigma Aldrich (USA). The anti-SOX9 (E-9, SOX9) and the anti-type I collagen (COLI) were obtained from Santa Cruz Biotechnology (USA). The biotinylated goat anti-mouse (IgG, H + L) and the avidin–biotin complex (ABC)- horseradish peroxidase (HRP) kit and Diaminobenzidine (DAB) Substrate Kit reagents were acquired from Vector Laboratories (USA).

### Parental and minicircle plasmids production and purification

**Fig. 1:**
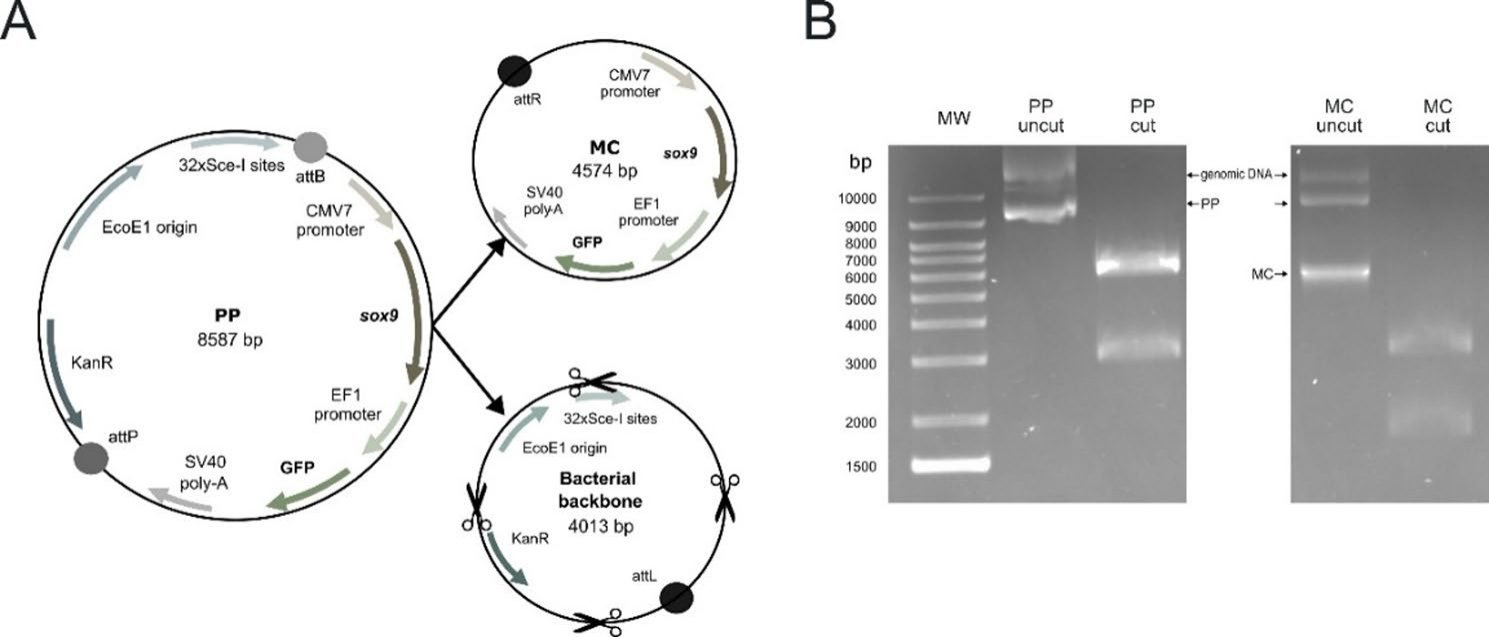
**Schematic representation of PP and MC plasmids with the corresponding agarose gel verification**. (**A**) DNA map composition of the parental plasmid (PP; MN511A-SOX9; pMC.CMV-MCS-EF1α-GFP-SV40poly A plasmid; 8587 bp), minicircle plasmid (MC; 4574 bp), and bacterial backbone (4013 bp). (**B**) Verification of both parental and minicircle plasmid by restriction enzyme treatment and agarose gel electrophoresis

The MN511A-SOX9 plasmid, defined as the parental plasmid GFP/SOX9 (PP; 8587 bp), was created by cloning SOX9 into the MN511A-1 vector (pMC.CMV-MCS-EF1α-GFP-SV40poly A plasmid) and served as the base to produce the minicircle GFP/SOX9 (MC; 4574 bp) (Fig. 1A).

MC was produced by the transformation of *E. coli* ZYCY10P3S2T minicircle producer strain with PP. Briefly, a single bacterial colony was picked and allowed to grow in 2 mL of LB medium containing 50 µg/mL kanamycin at 30 °C for 4–6 h with shaking at 250 rpm. Then, 1 mL of this culture was inoculated into a 1 L Erlenmeyer flask with 200 mL of 1X growth medium and incubated overnight at 37 °C with shaking at 250 rpm [26, 27]. Once the exponential growth phase was reached (OD600 4–8 and pH ~ 7), 1X induction media (200–400 mL) was added to the overnight culture before incubation for 4 h at 32 °C and 1 h at 37 °C to initiate the recombination process [26–28].

After verification by restriction enzyme digestion with SalI and XbaI and agarose gel electrophoresis, MC purification was performed using the HiPure Plasmid Maxiprep Kit following the manufacturer’s instructions, with suitable adaptations for minicircle isolation. A second verification step was conducted by electrophoresis as previously described. Simultaneously, PP was propagated in *E. coli* STLB2 and purified using the same kit, as aforementioned.

### Niosome preparation and nioplexe formation

Niosome formulations (DP20CQ and DP80CH) were synthesised by the thin-film hydration method [29], combining a fixed amount of the cationic lipid, DOTMA (2.5 mg; 1 mol ratio), with the non-ionic surfactants, P20 or P80 (2 mol ratio), and the corresponding helper lipids, CQ or CH (2 mol ratio), respectively. All components were first dissolved in dichloromethane (organic solvent) and thoroughly mixed. Then, the organic solvent was evaporated under argon flow to form a dried film at the bottom of the glass flask, followed by the addition of 2.5 mL of OptiMEM™ (aqueous medium) to resuspend the film. The solution was sonicated for 40 s at 50 W using the UP200S Sonifier (Hielscher Ultrasound Technology, Germany) [14].

Nioplexes were prepared by combining different volumes of niosomes with a specific volume of a stock solution of the corresponding plasmid (250 µg/mL) to achieve DOTMA/DNA mass ratios (w/w) of 5/1 and 10/1. The mixtures were allowed to stand for 30 min at room temperature to enhance electrostatic interactions between the positively charged cationic lipid, DOTMA and the negatively charged DNA.

### Size and zeta potential

Particle size and polydispersity index (PDI) were determined by Dynamic Light Scattering (DLS) using a Zetasizer Advance Range (Malvern, UK). Zeta potential determination by Electrophoretic Light Scattering (ELS) was carried out using the same instrument. Briefly, aliquots of the niosome and nioplexe formulations (150 µl) were filtered through 0.45-µm filters and resuspended in Milli-Q water previously filtered through 0.22-µm filters (final volume 800 µl). Folded capillary cuvettes (DTS0012, DTS1070) were employed for the measurements. All measurements were conducted in triplicate.

### Agarose gel electrophoresis

The ability of different niosome formulations (DP20CQ and DP80CH) to condense and protect both plasmids, PP and MC, from enzymatic degradation was assessed using an agarose gel electrophoresis assay [12]. Samples containing naked PP or MC (DOTMA/DNA ratio of 0) or complexed with niosomes (DOTMA/DNA ratios of 5/1 and 10/1, with 300 ng of plasmid in each case) were treated with 1 µl of DNase I (0.1 U/µl) [21]. The mixtures were incubated at 37 °C for 30 min, followed by the addition of 3 µL of 7% SDS solution to assess DNA release from the nioplexes [14]. Electrophoresis was performed on a 0.8% (w/v) agarose gel in TBE buffer at 80 V for 45 min. DNA bands were stained with SYBR Green Gold (0.01%) for 1 h, protected from light and stirred. The bands were visualised using a Chemi-Doc™ MP Imaging System (Bio-Rad, USA).

### Evaluation of niosome complexation capacity

A fluorescence-exclusion titration assay was used to assess the capacity of niosomes to bind and form complexes with a fixed amount of DNA [11]. Nioplexes were prepared as described in *Sect. 2.3*, maintaining a final volume of 50 µl. Following preparation, SYBR Green Gold (200X; 3 µL) was added, and the mixture was incubated in the dark at room temperature for 10 min. Subsequently, 10 mM HEPES was introduced to reach a total volume of 300 µl. Fluorescence was measured using a Synergy HTX Plate Reader (Biotek, USA) with black 96-well plates (λ_exc_ = 485 nm, λ_em_ = 528 nm). The fluorescence intensity (%) was calculated by normalizing the relative fluorescence units to the fluorescence of uncomplexed (naked) DNA, PP or MC, as described in Eq. 1:

$${\mathrm{Fluorescence~intensity~}}\left( {{\% }} \right)=\frac{{{{\mathrm{F}}_{{\mathrm{sample}}}}}}{{{{\mathrm{F}}_{{\mathrm{naked~PP~or~MC}}}}}}{\mathrm{~}} \times 100$$  

Where F_sample_ is the fluorescence recorded for each nioplexe formulation, and F_naked PP or MC_ corresponds to the fluorescence of the free plasmid in each case. Each condition was tested in triplicate. The complexation efficiency (%) was calculated by subtracting the fluorescence intensity (%) from 100, as shown in Eq. 2, reflecting the percentage of DNA plasmid complexed within the nioplexes:

$$ \begin{aligned}   Complexation~efficiency~\left( \%  \right) &  \\     = 100~ - ~Fluorescence~\mathrm{int} ensity~\left( \%  \right) \\  \end{aligned}  $$  

### TEM

Niosome dispersions (5 µL) were placed on carbon-coated grids and the excess was carefully removed with filter paper. Samples were dyed with 2% (v/v) uranyl acetate and observed using a JEM-1011 TEM (JEOL USA Inc., USA).

### hMSCs isolation and culture

Femoral head samples were obtained from patients undergoing hip arthroplasty (*n* = 2) and provided by the Biobanco of A Coruña. The study was approved by the Comité de Ética de Investigación da Coruña (accession number: 2021/425). All patients provided informed consent before inclusion in the study. Human MSCs (hMSCs) were isolated by flushing femoral heads with DMEM to collect bone marrow, filtering through a 70 μm cell strainer, and subsequently centrifuged at 1500 rpm for 5 min. The resulting cell pellet was resuspended in DMEM, 10% FBS, 1% P/S (growth medium). Cells were expanded in culture using standard protocols [30] and maintained in growth medium at 37 °C in a humidified atmosphere containing 5% CO_2_.

### hMSCs characterisation

Cells were trypsinised, washed and incubated at 4 °C for 45 min with antibodies: fluorescein isothiocyanate (FITC) isotype (1:50), phycoerythrin (PE) isotype (1:50), PE-conjugated anti-human CD34 (1:25), FITC-conjugated anti-human CD45 (1:25), PE-conjugated anti-human CD73 (1:25), FITC-conjugated anti-human CD90 (1:25) and FITC-conjugated anti-human CD105 (1:5). Following incubation, cells were washed and resuspended in PBS. Data acquisition was performed using a CytoFLEX flow cytometer (Beckman Coulter Life Sciences, Spain), and data analysis was conducted with CytExpert software (Beckman Coulter Life Sciences, Spain). A minimum of 10,000 cell events were acquired and analysed for each assay.

### Evaluation of GFP expression

For transfection experiments, cells were seeded in 48-well plates at a density of 4 × 10^4^ cells/cm^2^ and allowed to attach for 24 h at 37 °C. Then, cells were treated with nioplexes formed by complexing each niosome formulation, DP20CQ and DP80CH, with either PP or MC (always 0.32 µg/cm^2^ of plasmid per well; at DOTMA/DNA ratios of 5/1 and 10/1). Cells cultured in growth medium without nioplexes served as negative control, while cells transfected with the commercial reagent LPF (1 µL/ well) were used as positive control. hMSCs were further incubated for 24 h at 37 °C and 5% CO_2_ before being analysed by flow cytometry. All conditions were assessed in triplicate in at least two independent experiments.

The transfection efficiency was evaluated using flow cytometry. In brief, cells were washed with PBS, trypsinised, fixed, and washed again with several centrifugation steps. GFP expression was monitored using a CytoFLEX flow cytometer with data generated and processed by CytExpert software.

### Determination of SOX9 expression

hMSCs were seeded in 24-well plates (4 × 10^4^ cells/cm^2^) and allowed to attach 24 h at 37 °C. Subsequently, cells were exposed to the corresponding DP20CQ and DP80CH nioplexes. At 24 h post-transfection, cells were washed three times with cold PBS to remove residual medium. Then, 125 µl of a specific buffer (50 mM Tris, 0.9% NaCl, 0.1% SDS, pH 7.3) containing Halt™ protease and phosphatase inhibitor cocktail (1:100) was added per well to obtain the cell suspension. Following the manufacturer’s instructions, absorbance at 450 nm was measured using a Synergy HTX Plate Reader and the concentration of SOX9 (ng/mL) was calculated using a standard curve as reference.

### Assessment cell viability

Apoptosis was assessed by resuspending the cells in 5X annexin-binding buffer (diluted 1:5) containing Annexin V APC (1:1000), and Propidium Iodide staining solution (50 µg/mL; 1:100). Data acquisition and analysis were performed using CytExpert software using a CytoFLEX flow cytometer.

### Pellets formation

2 × 10^5^ hMSCs were placed in 1.5 mL conical tubes and centrifuged at 2000 rpm for 5 min to form 3D aggregate cultures [31, 32]. Pellets were then incubated in static conditions in serum-free chondrogenic medium (DMEM, 10 nM dexamethasone, 50 µg/mL ascorbic acid, 0.11 mg/mL pyruvate, 1x Insulin-Transferrin-Selenium A, 10 ng/mL TGF-β3) [33]. After 24 h, DP20CQ niosomes were complexed with PP or MC plasmids (1 µg plasmid; DOTMA/DNA ratio of 10/1) following the procedure described in *Sect. 2.3* and added to the hMSC pellets. Untransfected pellets cultured in chondrogenic medium or pellets transfected with the commercial reagent LPF, using the same amount of PP or MC plasmids (1 µg plasmid/pellet), were involved as negative and positive controls, respectively. All pellets were cultured for 21 days at 37 °C with media being changed every 3 days.

### Histological, immunohistochemical and histomorphometrical analyses

After 21 days of culture, hMSC aggregates were collected, fixed in 4% paraformaldehyde for 30 min, dehydrated in a graded ethanol series, and embedded in paraffin, following established protocols. Sections of 4 μm thickness were stained with 0.1% toluidine blue to detect sulphated proteoglycans and Harris Haematoxylin and 1% Eosin (H&E) for cell nuclei and cytoplasm.

Immunohistochemical analysis was carried out after blocking endogenous peroxidase using specific primary antibodies against SOX9 (1:100), type-I (1:200), type-II (1:10), and type-X collagen (1:200), followed by incubation with biotinylated secondary antibody using the ABC-HRP method with DAB as the chromogen [34].

Samples were analysed using a digital microscope (Lionheart FX, BioTek, USA). Histomorphometric analyses of histological and immunohistochemical images were performed using ImageJ. Measurements were taken from three randomly selected, standardised areas at 20x magnification for each experimental condition and replicate. Staining intensity was expressed as pixels per standardised area.

### Total RNA extraction and real-time RT-PCR analyses

Total cellular RNA was extracted from the cell aggregates using the RNeasy Protect Mini Kit. Reverse transcription was performed with 500 ng of the RNA eluate using the 1st Strand cDNA Synthesis Kit for RT-PCR (AMV). Subsequently, 10 ng of cDNA was used for amplification with real-time PCR employing the PowerUp™ SYBR™ Green Master Mix on QuantStudio 3 (Applied Biosystems, USA). The PCR conditions were set as follows: an initial denaturation from 50 °C to 95 °C at 1.6 °C/s for 2 min, followed by 50 cycles of denaturation at 95 °C for 15 s, annealing and extension at 60 °C for 1 min each. A final melting curve analysis was conducted from 95 °C to 65 °C at 1.6 °C/s and then to 95 °C at 0.5 °C/s. The primers used were specific for aggrecan (ACAN), a chondrogenic marker for proteoglycans (forward 5’-GAGATGGAGGGTGAGGTC-3’; reverse 5’-ACGCTGCCTCGGGCTTC-3’), SOX9, an early chondrogenic transcription factor (forward 5’-ACACACAGCTCACTCGACCTTG-3’), type-II collagen (COLII), a marker for chondrogenesis and matrix formation (forward 5’-GGACTTTTCTCCCCTCTCT-3’; reverse 5’-GACCCGAAGGTCTTACAGGA-3’), type-I collagen (COLI), an osteogenic marker (forward 5’-ACGTCCTGGTGAAGTTGGTC-3’; reverse 5’-ACCAGGGAAGCCTCTCTCTC-3’), and type-X collagen (COLX), a marker of hypertrophy (forward 5’-CCCTCTTGTTAGTGCCAACC-3’; reverse 5’-AGATTCCAGTCCTTGGGTCA-3’). GAPDH was used as a housekeeping gene and internal control (forward 5’-GAAGGTGAAGGTCGGAGTC-3’; reverse 5’-GAAGATGGTGATGGGATTTC-3’) [12], with all primers at a final concentration of 400 nM. The data were normalised to GAPDH expression, and results were calculated using the $\:{2}^{-\varDelta\:\varDelta\:Ct\:}$method.    

### Biochemical analyses

hMSC aggregates were digested with papain (25 µg, pH 6.5) at 60–65 °C for 1 h as previously described [13]. To estimate the proteoglycan content, dimethylmethylene blue (DMMB) dye was used with chondroitin sulfate C as the standard. Specifically, 250 µL of DMMB was added to each well containing diluted samples (40 µL in D-cysteine hydrochloride monohydrate solution, 1:1) and absorbance values were measured at 595 nm. Concentrations of proteoglycans of each sample were estimated using a calibration curve with chondroitin sulfate C.

Total protein was calculated by a bicinchoninic acid (BCA) assay using a calibration curve with BSA following the manufacturer’s instructions and measuring absorbance at 562 nm. Alternatively, DNA content was also evaluated by mixing samples (5 µl) with SYBR Green Gold (100X) and TE buffer, incubating for 10 min at room temperature before measuring fluorescence (λ_exc_ = 485 nm, λ_em_ = 528 nm). A calibration curve was prepared using type-I calf thymus. All measurements were performed with the aforementioned wavelengths in triplicate on a Synergy HTX Plate Reader.

### Statistical analyses

Experiments were assessed in at least two independent experiments from each donor and measurements were performed in triplicate. Data are expressed as mean ± standard deviation (SD). Statistical analyses were performed using parametric tests (two- and three-way ANOVA; Tukey’s test) and nonparametric ones (Kruskal-Wallis). *p* < 0.05 was considered statistically significant.

## Results

### Obtention and verification of minicircle from parental plasmid

The 4.6 kb minicircle plasmid was generated from an 8.6 kb parental construct using an arabinose-inducible system to express the ΦC31 integrase (Fig. 1A). This process results in the formation of the minicircle, while the bacterial backbone (4.0 kb) of the parental plasmid is subsequently removed through digestion by the I-SceI endonuclease, as denoted by agarose gel electrophoresis analyses (Fig. 1B).

### Physicochemical characterisation of niosomes and nioplexes

**Fig. 2:**
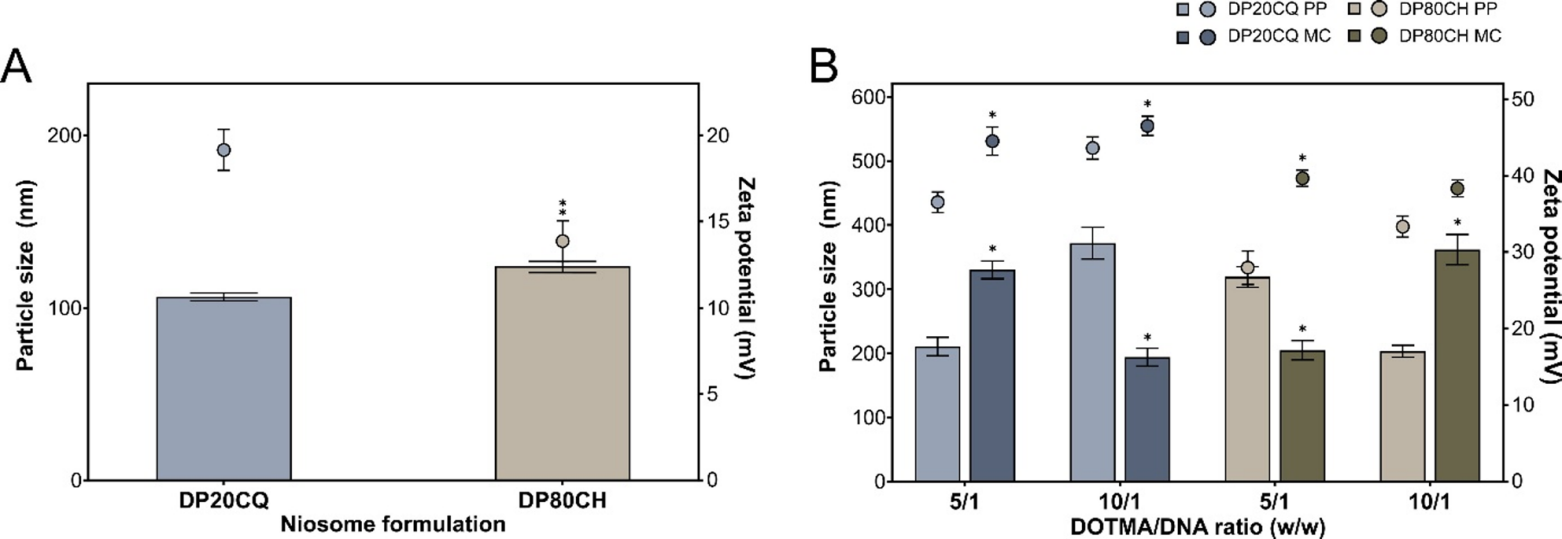
**Physicochemical characterisation of DP20CQ and DP80CH niosome and nioplexe formulations**. Particle size (bars) and zeta potential (symbols) of niosomes based on (**A**) polysorbate 20 and chloroquine (DP20CQ; blue) and polysorbate 80 and cholesterol (DP80CH; brown). * depicts *p* < 0.05, when compared both niosome formulations. (**B**) Particle size (bars) and zeta potential (symbols) of DP20CQ and DP80CH niosome formulations complexed with PP (light blue and light brown, respectively) and MC (dark blue and dark brown, respectively) at DOTMA/DNA ratio of 5/1 and 10/1. Data are shown as mean ± SD. * represents *p* < 0.05, when compared with the same niosome formulation complexed with different plasmids at a comparable DOTMA/DNA ratio (*n* = 3)

Both niosome formulations studied, DP20CQ and DP80CH, along with their respective nioplexes formed upon complexation with either plasmid (PP or MC), were characterised in terms of particle size (Figs. 2A and B), polydispersity index (PDI; Additional file 1, Table S1), and zeta potential (Figs. 2A and B).

Niosomes based on P20 and CQ (106.5 ± 2.4 nm) were significantly smaller than those composed of P80 and CH (124.0 ± 2.9 nm, *p* < 0.05) while also exhibiting a markedly higher electropositivity (19.2 ± 1.2 and 13.9 ± 1.2 mV, respectively; always *p* < 0.05; Fig. 2A).

In general, statistically significant differences were observed in terms of size and surface charge when compared the same niosome formulation at comparable DOTMA/DNA ratio for both plasmids (PP or MC; Fig. 2B). Mostly, DP20CQ niosomes complexed with MC (330.0 ± 13.6 nm) were larger than those combined with PP (210.8 ± 14.3 nm) at DOTMA/DNA ratio of 5/1 (*p* < 0.05), whereas the opposite trend was observed at DOTMA/DNA of 10/1 (194.1 ± 13.7 and 371.6 ± 25.0 nm, respectively; always *p* < 0.05). In contrast, DP80CH niosomes complexed with PP (319.1 ± 15.8 nm) were larger than MC complexes (204.9 ± 15.0 nm) at 5/1 DOTMA/DNA ratio (*p* < 0.05) but exhibited lower size at 10/1 DOTMA/DNA ratio (203.0 ± 9.5 and 361.4 ± 23.6 nm, respectively; *p* < 0.05). PDI values ranged between 0.31 and 0.48 for all niosomes and nioplexes (Additional file 1, Table S1).

Focusing on zeta potential values, both niosome formulations depicted the same tendency in surface charge. MC complexes (42.3 ± 1.6 mV) were consistently more electropositive than their PP counterparts (35.4 ± 1.3 mV; always *p* < 0.05), except those DP80CH nioplexes formed at a DOTMA/DNA ratio of 10/1, where this difference did not reach statistical significance (*p* = 0.24).

**Fig. 3:**
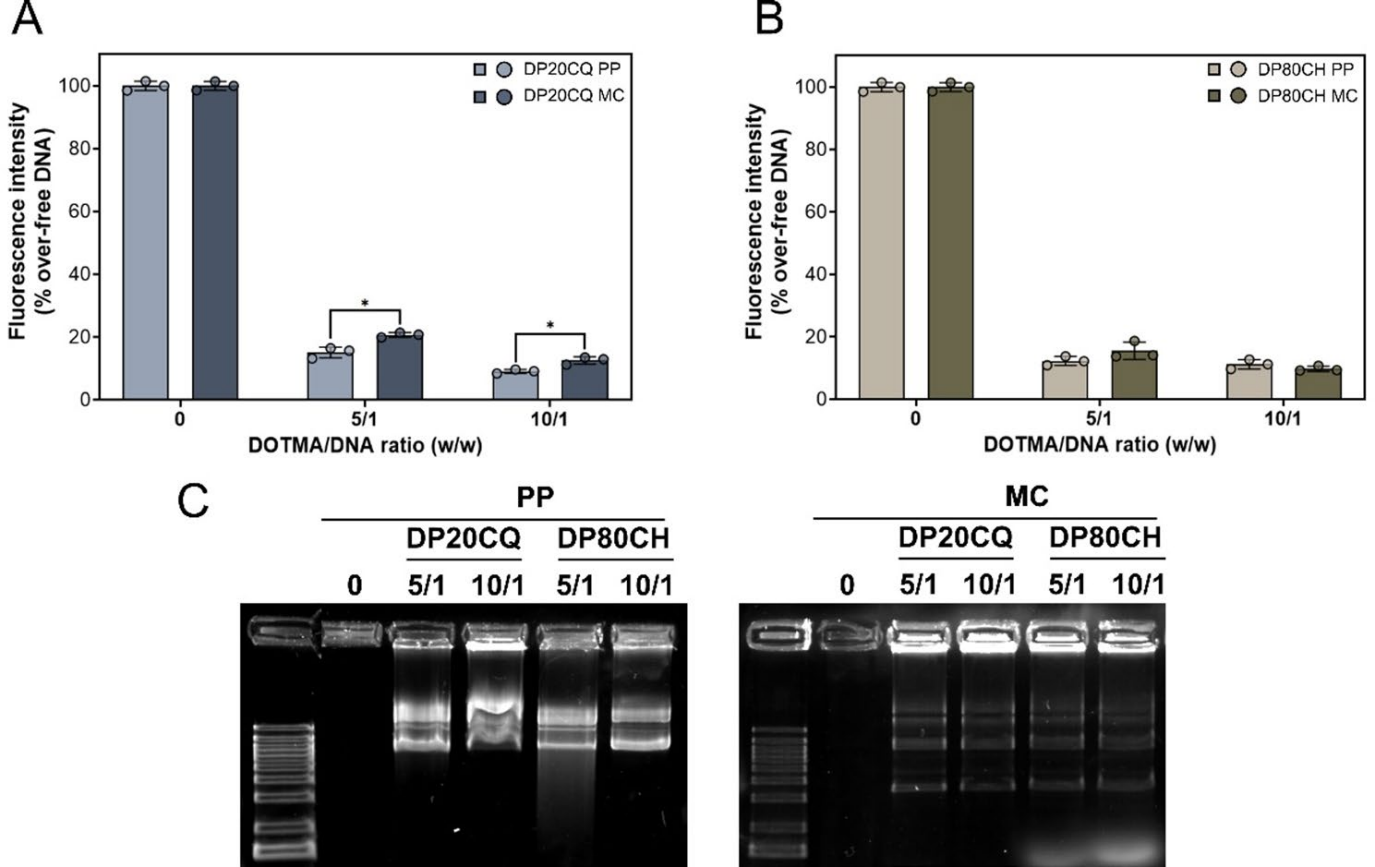
**Analysis of the complexation and protective properties of DP20CQ and DP80CH niosome formulations**. Fluorescence Intensity (%; **A** and **B**; bars) and DNase I protection ability and SDS-induced release visualised by agarose electrophoresis (**C**) of nioplexes based on polysorbate 20 and chloroquine (DP20CQ; **A**) and polysorbate 80 and cholesterol (DP80CH; **B**) with PP (light blue and light brown, respectively) and MC (dark blue and dark brown, respectively) at DOTMA/DNA ratio of 5/1 and 10/1. Data are shown as mean ± SD. * represents *p* < 0.05, when compared with the same niosome formulation complexed with different plasmids at the same DOTMA/DNA ratio. 0, naked PP and MC (*n* = 3)

The ability of both niosome formulations to efficiently condense PP and MC was evaluated using the SYBR Green Gold exclusion assay (Fig. 3A and B). Although a consistent decrease in fluorescence was noted with both DP20CQ and DP80CH-based nioplexes compared to naked PP or MC plasmids, different patterns were observed depending on the niosome composition. DP20CQ niosomes condensed PP more efficiently (~ 88%) than MC (~ 83%; always *p* < 0.05) at both DOTMA/DNA ratios (5/1 and 10/1) tested. Conversely, no significant differences were observed in complexation efficiency of DP80CH niosomes with either PP (~ 88%) or MC (~ 87%) at either of the two DOTMA/DNA ratios tested (*p* ≥ 0.24).

The capacity of DP20CQ and DP80CH formulations to protect DNA against DNase I degradation was also assessed by agarose gel electrophoresis (Fig. 3C). In contrast to that observed for free plasmid (0), an effective protection was observed at all DOTMA/DNA ratios, regardless of the niosome formulation or the plasmid used, as evidenced by the presence of bands corresponding to intact DNA, released from all nioplexe samples.

### In vitro transfection of hMSCs with niosome formulations

The surface marker profiles of hMSC primary cultures isolated from bone marrow aspirates were analysed (Additional file 2, Fig. S1A). Cells were positive for the three canonical MSC markers (CD90, CD73, and CD105; Additional file 2, Figs. S1B1 and B2). CD90 expression was > 99%, while CD105 and CD73 were expressed at lower levels (15 and 70%, respectively). In contrast, the expression of hematopoietic markers (CD45 and CD34; Additional file 2, Fig. S1B1 and B2, respectively) showed values ~ 3%.

The transfection efficiency of both niosome formulations complexed with either PP or MC was evaluated in primary hMSC cultures isolated from two different donors (donors #1 and #2) by flow cytometry.

**Fig. 4:**
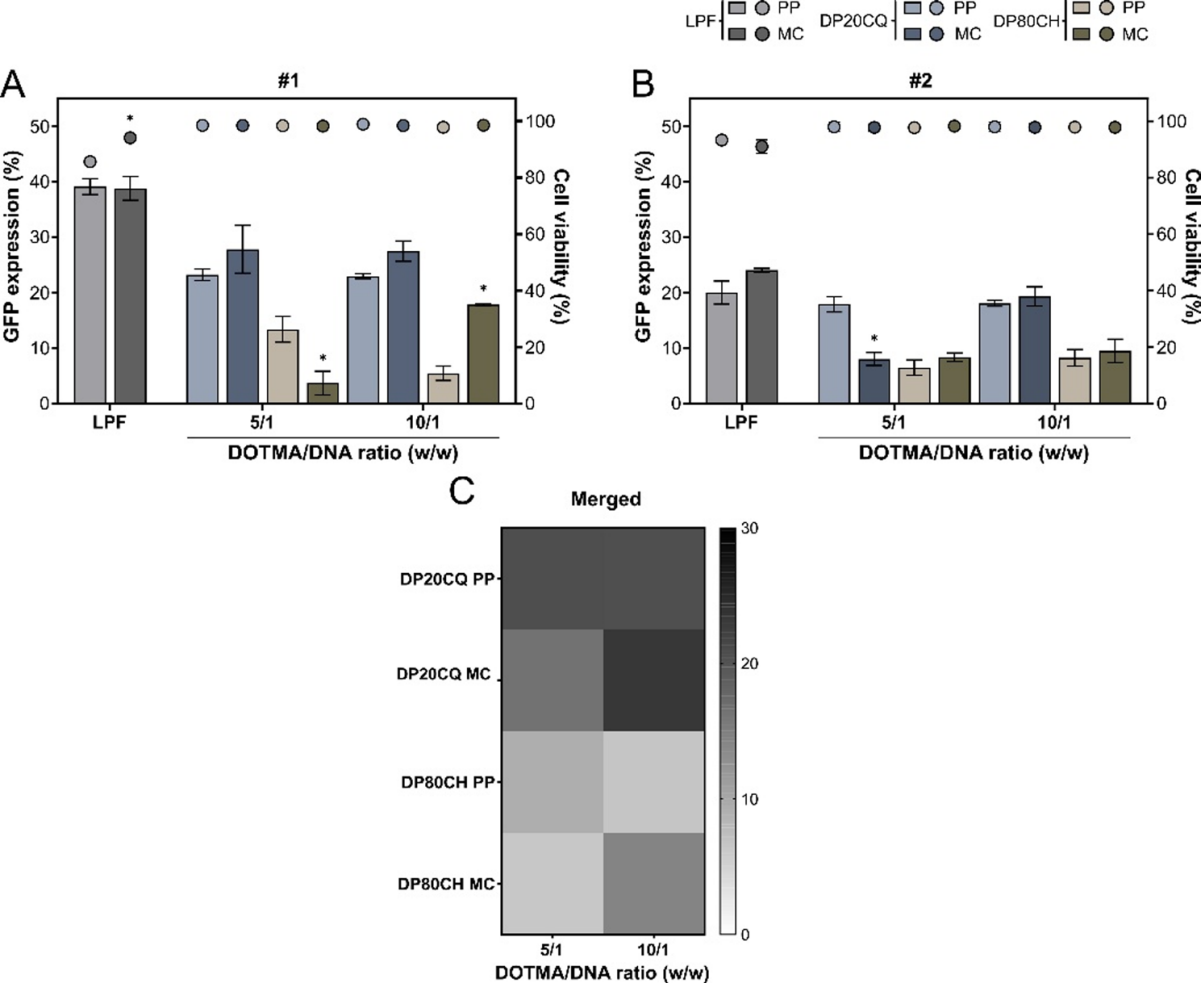
**Transfection efficiency of GFP via DP20CQ and DP80CH niosomes in hMSC monolayers**. GFP expression (%; bars) and cell viability (%; symbols) determined by flow cytometry after transfection of hMSC monolayers with Lipofectamine (LPF; grey) or niosomes based on polysorbate 20 and chloroquine (DP20CQ; blue) or polysorbate 80 and cholesterol (DP80CH; brown) complexed with parental plasmid (PP; light grey, light blue or light brown, respectively) or minicircle plasmid (MC; dark grey, dark blue or dark brown, respectively) in donor (**A**) #1 and (**B**) #2. Data are shown as mean ± SD. * depicts *p* < 0.05, when compared to the same formulation at a comparable DOTMA/DNA ratio with different plasmids (PP or MC) (*n* = 3). (**C**) Greyscale heatmap representing GFP expression levels (%) across two donors, where black denotes the highest and white the lowest transfection efficiency achieved (*n* = 6)

In both donors (Fig. 4), DP20CQ-based nioplexes achieved higher levels of GFP expression than those based on DP80CH (up to 3-fold increase). Moreover, in donor *#*1 (Fig. 4A), a slight increment was observed when DP20CQ was complexed with MC instead of PP, at both DOTMA/DNA ratios studied. A similar trend was noted in donor #2 (Fig. 4B) at a DOTMA/DNA ratio of 10/1.

In contrast, the type of plasmid played a more pronounced role in DP80CH-based nioplexes in donor #1, showing PP complexes consistently higher GFP expression at 5/1 DOTMA/DNA ratio (up to 3.6-fold difference; *p* ≤ 0.05), whereas MC complexes exhibited remarkably higher efficiency at 10/1 (up to 3.25-fold difference; *p* ≤ 0.05). In donor #2, DP80CH nioplexes had similar GFP percentages, regardless of the plasmid used. Notably, only the DP20CQ MC nioplexes at 10/1 DOTMA/DNA ratio achieved transfection levels not significantly different from those of the positive control, LPF, in both donors, with donor #2, showing absolute GFP expression values similar to those obtained with LPF.

A heatmap of merged donor data (Fig. 4C) revealed the same trend observed in individual donors, with the darkest grey, corresponding to the highest GFP expression, observed for DP20CQ complexed with MC, followed by PP, at a 10/1 DOTMA/DNA ratio. Furthermore, nioplexes formed with this same formulation at a 5/1 DOTMA/DNA ratio also led to higher GFP intensities compared to those observed for DP80CH-based nioplexes.

An analysis of cell viability showed high levels of cell survival for all nioplexe formulations (Figs. 4A and B), reaching up to 97.8% in donor #1 and 98.4% in donor #2. Of note, in all cases, niosomes exhibited significantly lower cytotoxicity compared to LPF, irrespective of the plasmid used, never dropping below 94.1% in either donor (*p* ≤ 0.05).

**Fig. 5:**
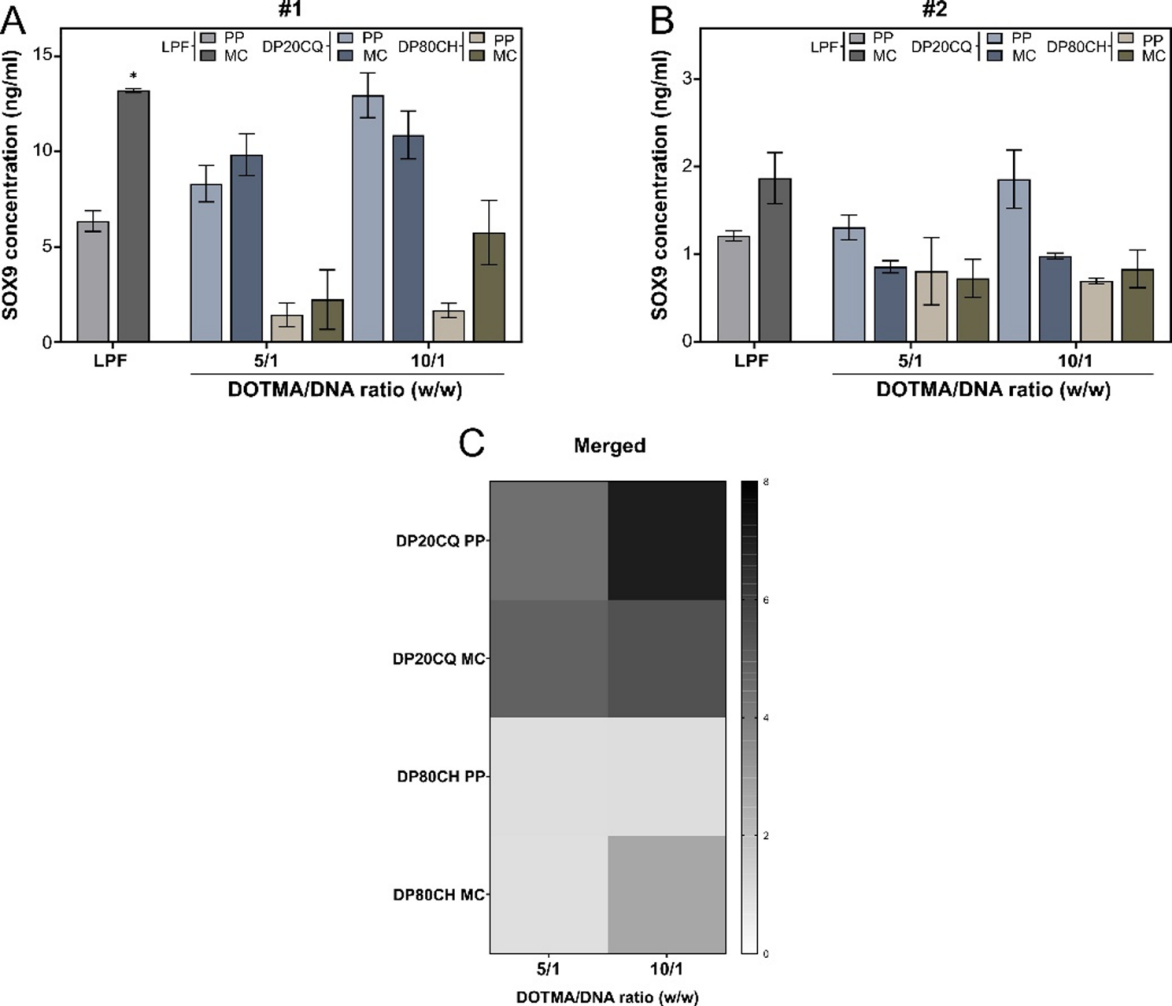
**SOX9 transfection efficiency of DP20CQ and DP80CH niosomes in hMSC monolayers**. SOX9 concentrations (ng/mL; bars) determined by ELISA after transfection of hMSC monolayers with Lipofectamine (LPF; grey) or niosomes based on polysorbate 20 and chloroquine (DP20CQ; blue) or polysorbate 80 and cholesterol (DP80CH; brown) complexed with parental plasmid (PP; light grey, light blue or light brown, respectively) or minicircle plasmid (MC; dark grey, dark blue or dark brown, respectively) in donor (**A**) #1 and (**B**) #2. Data are shown as mean ± SD. * depicts *p* < 0.05, when compared to the same formulation at a comparable DOTMA/DNA ratio with different plasmids (PP or MC) (*n* = 3). (**C**) Greyscale heatmap representing SOX9 concentration (ng/mL) across two donors, where black denotes the highest and white the lowest levels of expression (*n* = 6)

Studies on SOX9 expression by ELISA revealed a similar pattern to that observed for GFP transgene expression in cells isolated from both donors (Fig. 5). DP20CQ nioplexes consistently yielded higher SOX9 concentrations than DP80CH nioplexes, with no significant differences compared to the positive control, LPF. Notably, in donor #1 (Fig. 5A), DP20CQ at a DOTMA/DNA ratio of 10/1 achieved the highest SOX9 expression levels, showing values similar to those obtained with LPF, regardless of the plasmid used. In contrast, in donor #2 (Fig. 5B), only the PP complexes yielded comparable results. In any case, DP80CH nioplexes only exhibited results as efficient as the DP20CQ ones in donor #2. Of note, a significantly higher transfection was observed for LPF when using MC lipoplexes compared to their PP counterparts.

A greyscale heatmap of merged SOX9 expression data from both donors (Fig. 5C) depicted the superior ability of DP20CQ to genetically modify hMSCs compared to DP80CH. Moreover, in all cases, a DOTMA/DNA ratio of 10/1 demonstrated higher transfection efficiency than the 5/1 ratio.

### Chondrogenic differentiation of hMSCs upon transfection with nioplexes

**Fig. 6:**
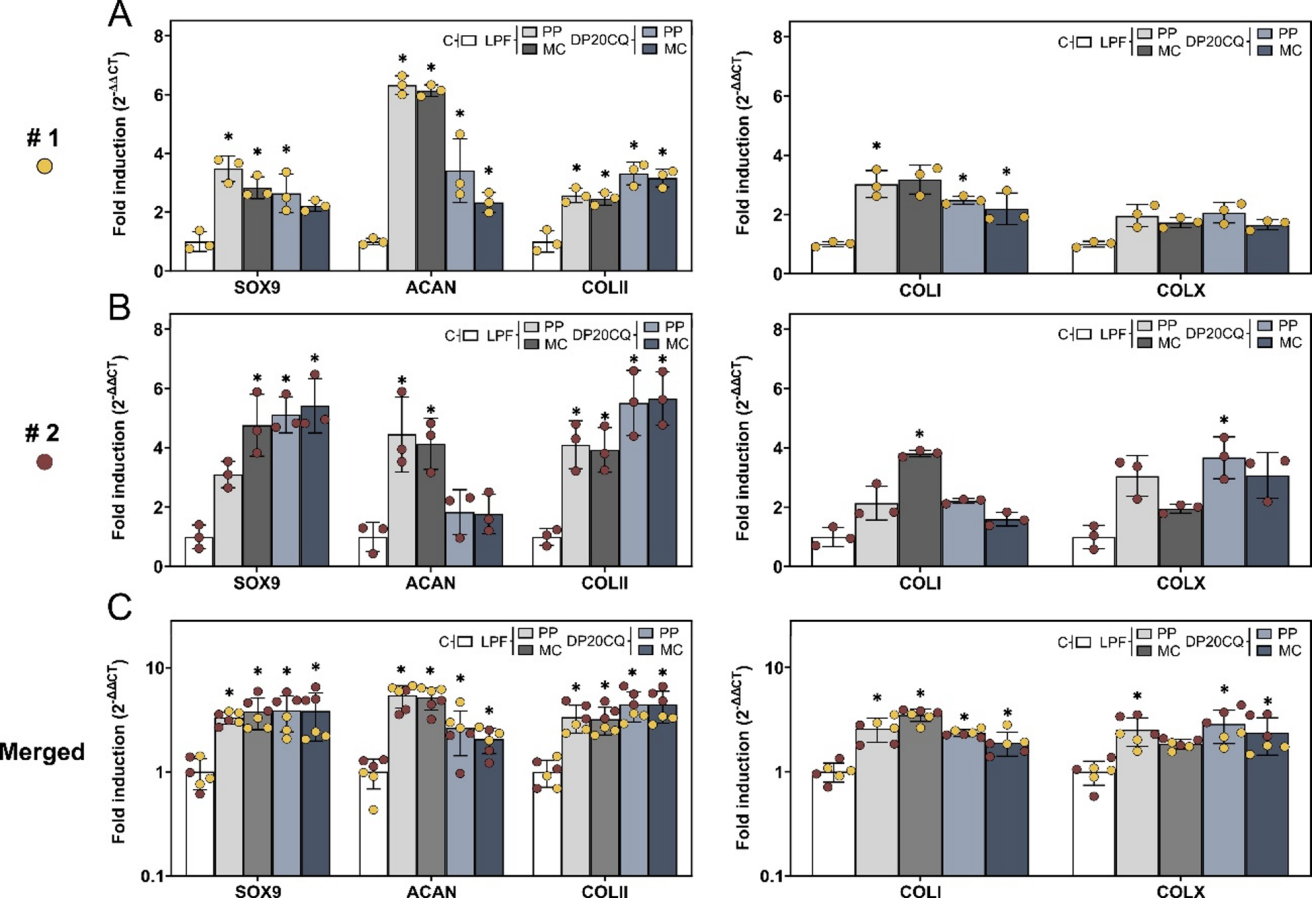
**qPCR analyses of chondrogenesis**. RT-qPCR analysis (bars) of hMSC aggregates from two donors (donor #1, dark yellow, **A**; donor #2, dark red, **B**; merged, **C**) and cultured in vitro 21 days in chondrogenic medium (negative control; white) and transfected with Lipofectamine (LPF; grey) or niosomes based on polysorbate 20 and chloroquine (DP20CQ; blue) and complexed with either parental plasmid (PP; light grey or light blue, respectively) or minicircle (MC; dark grey or dark blue, respectively) containing SOX9. (**A-C**, left panels) Expression of the SOX9 transgene and its downstream chondrogenic markers, such as aggrecan (ACAN) and type-II collagen (COLII), is shown in the left panels. (**A-C**, right panels) Expression of hypertrophy markers such as type-I (COLI) and type-X collagen (COLX), is represented in the right panels. Ct values were normalised to GADPH, and fold inductions were calculated considering 2^-ΔΔCt^ method relative to the negative control. * depicts *p* < 0.05 when compared to the negative control (**A-B**, *n* = 3; **C**, *n* = 6)

**Fig. 7:**
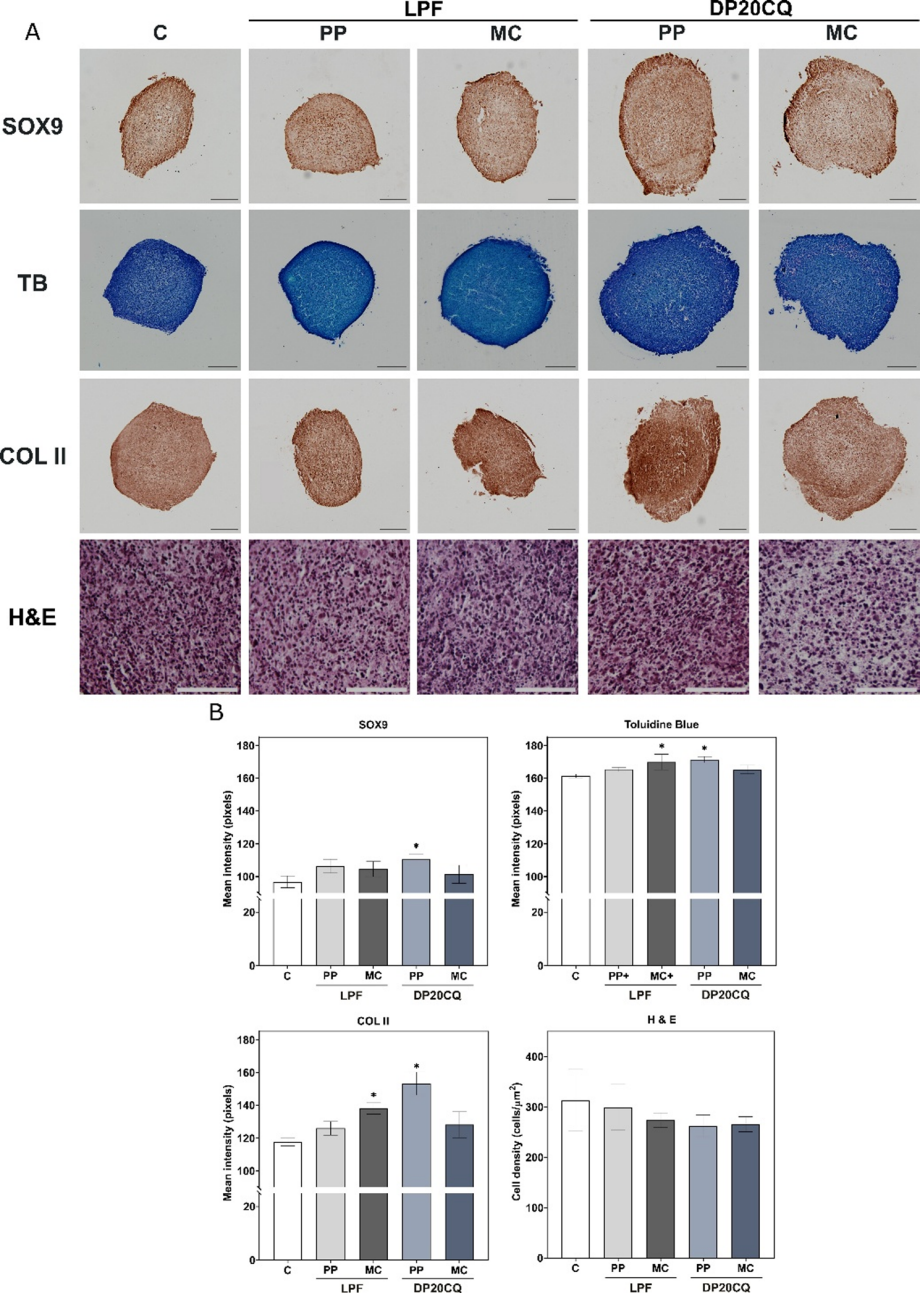
**Immunohistochemical and histological analyses of chondrogenic markers**. Immunohistochemical and histological analyses of hMSC aggregates from donor #2 cultured in vitro for 21 days in chondrogenic medium (negative control, C; white) and transfected with Lipofectamine (LPF; grey) or niosomes based on polysorbate 20 and chloroquine (DP20CQ; blue) and complexed with either parental plasmid (PP; light grey or light blue, respectively) or minicircle (MC; dark grey or dark blue, respectively). (**A**) Immunodetection of SOX9, toluidine blue (TB), type-II collagen (COLII; magnification 10x; scale bar 200 μm); and Haematoxylin/Eosin (H&E) (magnification 40x; scale bar: 100 μm). (**B**) Histomorphometric quantification (bars) corresponds to the images shown. Data are shown as mean ± SD. * depicts *p* < 0.05, when compared to the negative control (*n* = 3)

**Fig. 8:**
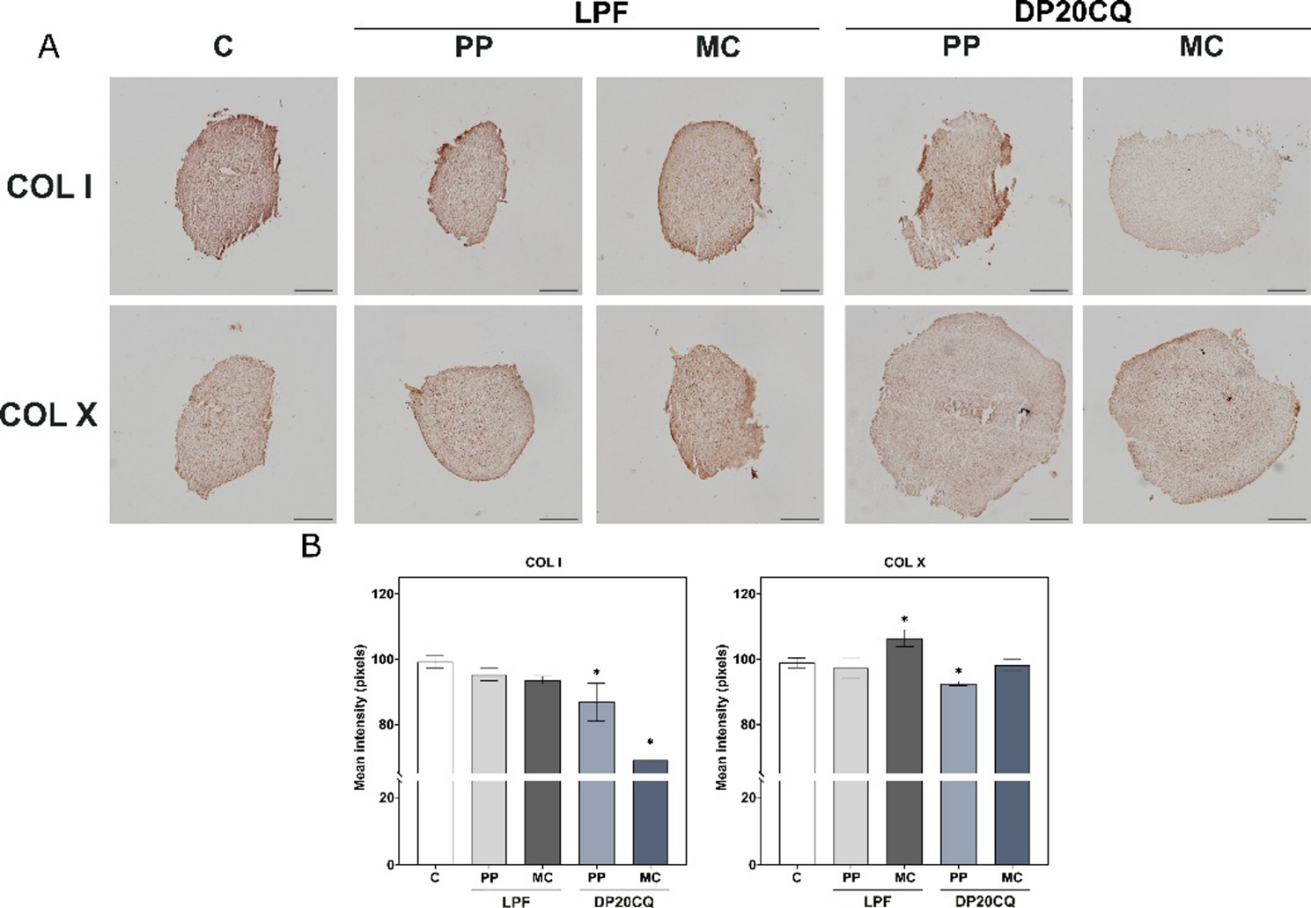
**Immunohistochemical and histological analyses of hypertrophic markers**. Immunohistochemical and histological analyses of hMSC aggregates from donor #2 cultured in vitro for 21 days in chondrogenic medium (negative control, C; white) and transfected with Lipofectamine (LPF; grey) or niosomes based on polysorbate 20 and chloroquine (DP20CQ; blue) and complexed with either parental plasmid (PP; light grey or light blue, respectively) or minicircle (MC; dark grey or dark blue, respectively). (**A**) Immunodetection of type-I (COLI) and type-X collagen (COLX) (magnification 10x; scale bar: 200 μm). (**B**) Histomorphometric quantification (bars) corresponds to the images shown. Data are shown as mean ± SD. * depicts *p* < 0.05, when compared with the negative control (*n* = 3)

**Fig. 9:**
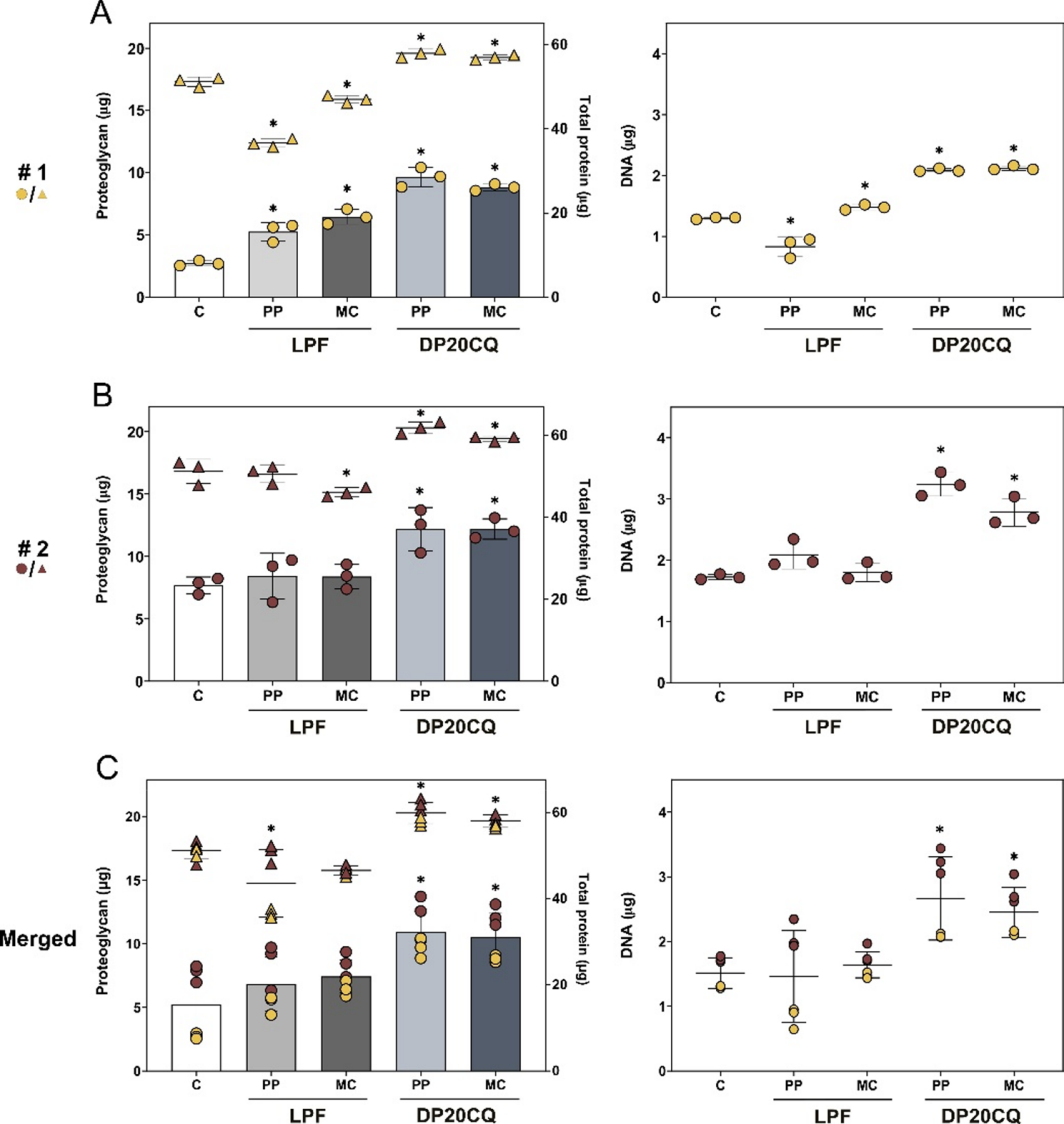
**Biochemical analyses of chondrogenesis**. Proteoglycans (µg; **A-C**, left panels; bars), proteins (µg; **A-C**, left panels; symbols) and DNA (µg; **A-C**, right panels; symbols) profiles of hMSC aggregates from two donors (donor #1, dark yellow, **A**; donor #2, dark red, **B**; merged, **C**) cultured in vitro for 21 days in chondrogenic medium (negative control, C; white) and transfected with Lipofectamine (LPF; grey) or niosomes based on polysorbate 20 and chloroquine (DP20CQ; blue) and complexed with either parental plasmid (PP; light grey or light blue, respectively) or minicircle (MC; dark grey or dark blue, respectively). Data are shown as mean ± SD. * depicts *p* < 0.05 compared to DP20CQ and LPF complexes with negative control (**A-B**, *n* = 3; **C**, *n* = 6)

Once the most optimal niosome composition was determined (DP20CQ at a 10/1 DOTMA/DNA ratio), its efficacy in promoting chondrogenesis of hMSCs was investigated in a 3D aggregate culture model by delivering PP and MC plasmids containing the transcription factor SOX9. Cell pellets were cultured for 21 days in chondrogenic medium before analysis. Subsequent evaluations included RT-qPCR (Fig. 6), as well as histological, immunohistochemical, histomorphometrical (Figs. 7 and 8), and biochemical analyses (Fig. 9).

A RT-qPCR analysis of SOX9 expression showed a successful transfection of SOX9 in hMSC aggregates transfected with DP20CQ or LPF complexes, with either PP or MC plasmids compared to untransfected negative control group (Fig. 6). Notably, the levels of SOX9 expression achieved with DP20CQ-based nioplexes did not show statistically significant differences from those obtained with LPF for both PP and MC plasmids (*p* > 0.05), with this trend being more pronounced in donor #2 (Fig. 6B, left panel) than in donor #1 (Fig. 6A, left panel). Histomorphometric analysis of SOX9 immunostaining (Fig. 7) for hMSCs from donor #2 corroborated gene expression data, revealing always higher mean staining intensity in all transfected groups relative to the negative control (96.8 ± 3.6 pixels), although statistical significance was only reached with DP20CQ complexed with PP (110.5 ± 3.0 pixels, *p* < 0.05).

Chondrogenic differentiation was evidenced in every condition, as demonstrated by the upregulation of aggrecan (ACAN) and type-II collagen (COLII) in both donors (Fig. 6C, left panel). In donor #1 (Fig. 6A, left panel), ACAN expression increased across all conditions (with both plasmids and formulations), whereas in donor #2 (Fig. 6B, left panel), a significant rise was detected only in LPF‑transfected pellets (*p* < 0.05). Histological analysis of cartilage extracellular matrix by toluidine blue staining (Figs. 7A and B) revealed higher proteoglycan deposition in all transfected conditions compared to cell pellets cultured in chondrogenic medium solely (161.1 ± 0.2 pixels). Notably, DP20CQ complexed with PP (169.9 ± 3.0 pixels) and LPF complexed with MC (171.9 ± 5.7 pixels) led to the highest toluidine blue mean intensities (*p* < 0.05).

The expression of COLII was significantly higher in DP20CQ complexes than in their LPF counterparts in both donors (Figs. 6A and B, left panel, *p* < 0.05). COLII immunoreactivity analysis (Figs. 7A and B) corroborated these findings, leading DP20CQ complexed with PP to the highest intensities (up to 1.3-fold difference compared with negative control; *p* < 0.05).

H&E staining (Figs. 7A and B) revealed slightly reduced cell density in pellets transfected with DP20CQ or LPF complexes compared with the negative control, although no statistically significant differences were observed (*p* > 0.45).

The expression of the hypertrophic markers, type-I collagen (COLI) and type-X collagen (COLX) was slightly upregulated in transfected aggregates when compared to the negative control, in cells from both donors (Figs. 6A and B, right panel). Of note, COLI upregulation was higher in pellets transfected with LPF lipoplexes compared to their DP20CQ counterparts with either PP or MC plasmids, and in hMSCs from both donors (Figs. 6A and B, right panel). An analysis of COLI immunoreactivity (Figs. 8A and B) revealed a statistically significant reduction in this marker upon transfection with DP20CQ, with the MC-based formulation showing the most pronounced decrease (*p* < 0.05).

Focusing on the expression of COLX, comparable results were obtained for all conditions for donor #1 (Fig. 6A, right panel), while a slight increase in this marker was revealed in cell aggregates from donor #2 transfected with DP20CQ using PP plasmid (Fig. 6B, right panel). A similar tendency was observed in those cell aggregates transfected with the same plasmid using LPF, when data from both donors were considered (Fig. 6C). In contrast, analysis of COLX immunoreactivity (Figs. 8A and B), showed the highest intensities with cell pellets transfected with LPF/MC complexes, while a significant reduction was observed following transfection with DP20CQ carrying PP plasmid (*p* < 0.05).

These findings were further supported by biochemical analysis of proteoglycan content, protein levels, and DNA profiles in hMSC aggregate cultures (Fig. 9).

Overexpression of SOX9 in hMSC aggregates via DP20CQ niosomes always led to the highest proteoglycan deposition (up to 2.1-fold difference) when compared to pellets cultured in chondrogenic medium alone, regardless of the type of plasmid used (PP or MC) or the cell donor studied (Figs. 9A-C, left panels). An increase in proteoglycan content was also evidenced upon transfection with LPF lipoplexes, although statistically significant differences were only reached in cells from donor #1 (*p* < 0.05; Fig. 9A, left panel).

Beyond, cell aggregates transfected with LPF complexed with PP or MC exhibited significant reductions in both protein (43.6 ± 7.8 µg and 46.5 ± 1.0 µg, respectively; Figs. 9A-C, left panels) and DNA contents (1.5 ± 0.7 µg and 1.6 ± 0.2 µg, respectively; Figs. 9A-C, right panels) when compared to those transfected with DP20CQ complexed with PP (59.9 ± 2.4 µg and 2.7 ± 0.6 µg, respectively) or MC (58.1 ± 1.4 µg and 2.5 ± 0.4 µg, respectively). No significant differences were detected between PP and MC when delivered with the same carrier formulation (*p* ≥ 0.07).

**Fig. 10:**
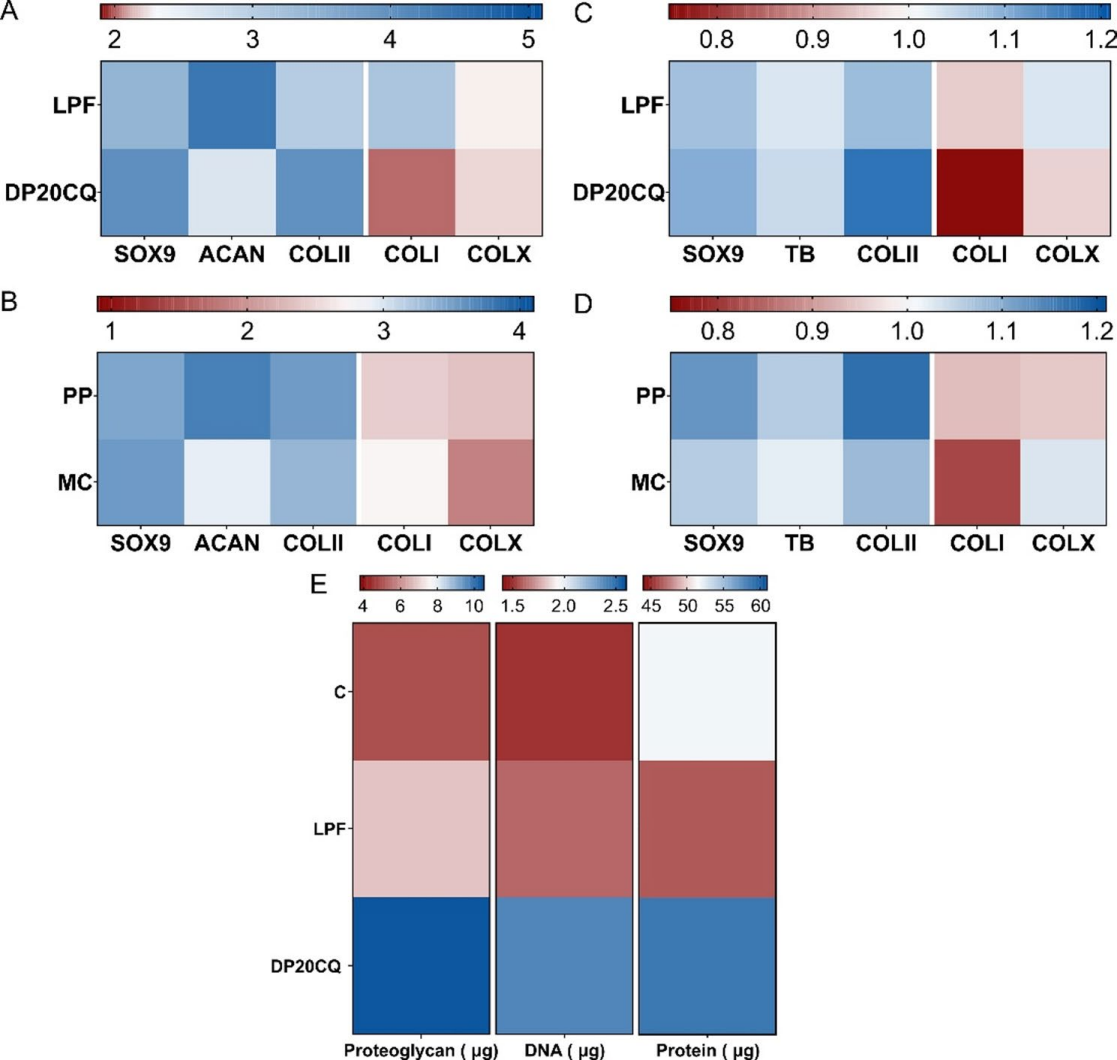
**Heatmap of chondrogenic and hypertrophic markers classified by vector type and plasmid used**. Heatmap representing the expression of chondrogenic (SOX9, ACAN, COLII) and hypertrophic and osteogenic markers (COLI and COLX) measured by qPCR analyses of hMSC aggregates from both donors transfected with (**A**, upper panel) Lipofectamine (LPF) or niosomes based on polysorbate 20 and chloroquine (DP20CQ) and complexed with either (**B**, lower panel) parental plasmid (PP) or minicircle (MC) relative to the untransfected control group (*n* = 12). Heatmap representing histomorphometric changes of hMSC aggregates from donor #2 transfected with (**C**, upper panel) Lipofectamine (LPF) or niosomes based on polysorbate 20 and chloroquine (DP20CQ) and complexed with either (**D**, lower panel) parental plasmid (PP) or minicircle (MC) relative to the untransfected control group (*n* = 6). (**C**) Heatmap representing biochemical analysis of hMSC aggregates from both donors, untransfected (**E**) or transfected with Lipofectamine (LPF) or niosomes based on polysorbate 20 and chloroquine (DP20CQ) (*n* = 12). Blue denotes the highest and red the lowest values

To illustrate the influence of vector and plasmid type on the chondrogenesis efficiency of MSCs, a heatmap representing expression levels and histomorphometric changes in key chondrogenic markers, as well as extracellular matrix biochemistry analysis relative to the untransfected control group, was constructed (Figs. 10A–D). Focusing on vector type (Figs. 10A and C, upper panels), transfection with DP20CQ niosome formulation simultaneously increased the expression of key chondrogenic markers while reducing the expression of hypertrophic markers. A similar trend was observed when studying the influence of the genetic cargo (Figs. 10B and D, lower panels), showing that the use of the PP elicited the most desirable features in hMSC chondrogenesis. Lastly, a heatmap representation of the biochemical composition (Fig. 10E) pointed out the influence of the formulation, with DP20CQ complexes leading to a higher production of both protein and DNA content compared to LPF or control groups.

## Discussion

Human mesenchymal stem cells (hMSCs) are extensively investigated for clinical applications in both cell- and gene-based therapies owing to their ease of isolation from various adult tissues and their inherent multipotency [35]. Their ability to differentiate into chondrogenic, osteogenic, and adipogenic lineages makes them attractive candidates for a broad spectrum of regenerative applications related to cartilage and bone lesions. Still, their therapeutic efficacy is often hindered by intrinsic limitations, including reduced production of paracrine factors, and the detriment of their proliferative and differentiation capacities with ageing [36]. As a result, hMSCs frequently fail to regenerate native articular hyaline cartilage, instead promoting the development of undesired fibrocartilaginous or hypertrophic tissue, which lacks the biomechanical properties of the cartilage found in the joint [1]. Hence, gene therapy has emerged as a robust strategy to overcome these shortcomings and enhance the clinical efficacy of hMSCs.

To this end, non-viral strategies have gained increasing attention, as they circumvent the major drawbacks associated with viral vectors, including immunogenicity and insertional mutagenesis. However, their clinical application remains constrained by low transfection efficiency and limited cell specificity [37, 38]. These challenges become even more pronounced when transfecting primary cells such as hMSCs, where multiple biological barriers such as uptake, endosomal escape, and nuclear entry must be overcome while minimising transfection-related cytotoxicity [39].

In an effort to address these limitations, current strategies have focused on the rational design of the delivery vector and the optimisation of the genetic cargo composition [39, 40]. In this context, among the various non-viral platforms under investigation, niosomes have come to the forefront of gene delivery as promising candidates due to their biocompatibility, low immunogenicity, and tunable physicochemical properties. Previous studies from our group have screened a library of niosomes to identify optimal compositions and mole ratios to transfect immortalised MSCs. Our findings showed that those formulations composed of the cationic lipid, DOTMA (D), with the non-ionic surfactants, polysorbate 20 (P20) or 80 (P80) and the helper lipids, chloroquine (CQ; DP20CQ) or cholesterol (CH; DP80CH), led to the highest transfection efficiencies in iMSCs [14].

To further optimise the genetic cargo, minicircle vectors have emerged as a promising alternative to conventional parental plasmids, offering improved transfection efficiency and sustained transgene expression due to the absence of bacterial backbone elements [25]. These minimal, supercoiled DNA constructs contain only the therapeutic expression cassette, resulting in enhanced and prolonged transgene expression [23–25]. In line with this, recent studies have demonstrated their potential to drive chondrogenic differentiation in MSC-like cells, underscoring their relevance for cartilage regenerative approaches [41, 42].

Based on this, in the present work, we evaluated the performance of the most promising niosome formulations (DP20CQ and DP80CH) to transfect primary bone marrow-derived hMSCs. To assess the impact of the genetic cargo, these systems were used to deliver either a conventional parental plasmid (PP) or its corresponding DNA minicircle (MC), both encoding for GFP and SOX9.

Physicochemical characterisation of niosomes and their corresponding nioplexes formed with either PP or MC confirmed the successful formation of stable nanostructures within the optimal range for cellular uptake, from 100 to 400 nm [14, 43]. Notably, the surface charge of both PP and MC complexes reached values of up to 20 mV, promoting colloidal stability by preventing particle aggregation and enhancing cellular internalisation via electrostatic interactions with the negatively charged sulphated proteoglycans on the cell surface [43]. It is worth mentioning that DP20CQ niosomes displayed higher zeta potential values than their DP80CH counterparts. This tendency is likely attributable to the presence of chloroquine, responsible for enhancing bilayer’s fluidity and facilitating electrostatic condensation of nucleic acids [43]. In concordance with these findings, both DP20CQ and DP80CH formulations demonstrated the capacity to efficiently condense either PP or MC genetic cargo while protecting them from DNase degradation, as evidenced by the presence of respective bands in the agarose gel electrophoresis [14, 44].

An analysis of GFP reporter gene expression revealed that the DP20CQ formulation, complexed with either PP or MC plasmids, consistently yielded higher transfection efficiencies than the DP80CH-based complexes. This result contrasts with previous studies in immortalised MSCs, where transfection outcomes of DP20CQ and DP80CH were comparable [14], suggesting that cell–type–specific differences may underlie the enhanced performance of DP20CQ in primary MSCs. Immortalised cells typically exhibit altered membrane composition, increased proliferation, and enhanced endocytic activity, which can facilitate nanoparticle uptake and intracellular trafficking [45]. In contrast, primary MSCs are more resistant to genetic modification and display rapid endosomal maturation, making efficient endosomal escape a more critical determinant of successful transfection. In this context, previous studies have described a significantly enhanced intracellular trafficking and transgene expression when using niosomes based on polysorbate 20 (P20) compared to those containing polysorbate 80 (P80) or 85, in retinal pigment epithelial cells (ARPE-19) [46]. This improvement is likely related to the higher hydrophilic-lipophilic balance (HLB) of P20 (HLB = 16.7) relative to P80 (HLB = 15), promoting an increased colloidal stability and more efficient cellular uptake [46, 47]. In addition, this effect may be further potentiated by chloroquine, previously shown to enhance endosomal escape [43].

Despite their theoretical advantages [41, 42, 48], minicircle vectors exhibited transfection efficiencies in hMSCs comparable to their parental plasmid, regardless of the delivery system used (DP20CQ, DP80CH, or LPF). Importantly, both vectors were delivered at the same DNA mass, a common practice in transfection studies that allows direct comparison between constructs of different sizes. Since the minicircle (4.6 kb) is approximately half the length of the parental plasmid (8.6 kb), this normalisation by mass resulted in a higher molar dose of expression cassette in the minicircle group. DOTMA content in the niosome formulations was calculated based on DNA mass and adjusted to achieve the targeted lipid-to-DNA weight ratios (5/1 and 10/1, w/w) for both plasmids. This approach ensured that both parental and minicircle vectors were tested under identical conditions, following standard lipid–nucleic acid complexation protocols, where the DOTMA-to-DNA mass reflects the intended N/P ratio (as the number of phosphate groups per unit mass of DNA remains constant). However, because minicircles contain more DNA molecules per unit mass, the effective lipid-to-DNA molar ratio was consequently lower, which may have led to suboptimal complex formation and reduced transfection performance. Future studies incorporating molar normalisation or vector copy number adjustment may help clarify these observations. In addition, residual contamination with parental DNA plasmid following minicircle production [42, 48] may potentially impair transfection efficiency, thereby increasing the variability of the results. Similar findings were reported by Kozisek et al. [37], where minicircles consistently yielded lower transfection efficiency and transgene expression than conventional plasmids under normalised experimental conditions. In light of this, further purification steps such as fast protein liquid chromatography (FPLC) may be required to ensure the exclusive presence of minicircle DNA [49, 50].

It is noteworthy that the highest levels of transfection achieved with DP20CQ niosomes were observed when assessing SOX9 expression by ELISA, with values that were not statistically different from those achieved with the commercial reagent LPF. This difference may reflect the use of distinct promoters regulating the reporter and the therapeutic gene expression, being CMV and EFα1 promoters for SOX9 and GFP, respectively. Along these lines, previous studies have reported a significantly increased transcriptional activity driven by CMV compared to EF1α promoter, particularly in hMSCs [37].

After selecting the DP20CQ niosomes formulation (DOTMA/DNA ratio 10/1) as the most suitable for transfection of hMSCs, we evaluated its performance in delivering the transcription factor SOX9 in a 3D aggregate culture model, aiming to recapitulate key features of the native cartilage microenvironment and rigorously assess the chondrogenic efficacy of SOX9 gene delivery [51, 52].

As a matter of fact, niosomes have previously demonstrated a promising capacity to promote chondrogenic differentiation of hMSCs in a similar model [13, 41]. However, in this work, niosomes failed to produce similar levels of chondrogenic markers expression achieved with LPF at both transcriptional and protein levels [13]. In our study, chondrogenesis was confirmed across all experimental groups, as evidenced by the expression of SOX9, deposition of sulphated glycosaminoglycans and production of type-II collagen, hallmarks of hyaline-like cartilage tissue.

The sex-determining region Y-type high-mobility group box 9 (SOX9) is well-established as a master regulator of chondrocyte differentiation and cartilage formation, not only driving the initial stages of chondrogenic differentiation but also maintaining the chondrocyte phenotype while suppressing hypertrophic and osteogenic markers that could compromise cartilage integrity [1, 13, 33].

Overexpression of SOX9 via DP20CQ complexed with PP significantly upregulated key extracellular matrix components of hyaline cartilage, such as aggrecan (ACAN) and type-II collagen (COLII), while repressing type-I (COLI) and type-X (COLX) collagen, markers associated with fibrocartilage and hypertrophy phenotypes [13]. These results highlight the synergistic effect of SOX9 beyond TGF-β supplementation, enhancing COLII and ACAN expression while attenuating COLI and COLX in selected conditions [1, 13, 53]. Nevertheless, in line with previous reports, COLI and COLX were still slightly upregulated in some conditions, even in DP20CQ-transfected groups, reflecting the natural propensity of bone marrow–derived MSCs to undergo hypertrophic differentiation [54]. This finding supports the view that additional molecular cues may be necessary to restrain hypertrophic differentiation in bone marrow–derived MSCs [55].

In contrast, overexpression of SOX9 via LPF not only resulted in markedly weaker staining of chondrogenic markers but also induced a notable upregulation and increased staining of COLI and COLX [13]. These observations support the idea that LPF may entail MSCs behaviour in favour of a hypertrophic differentiation [12, 55].

Focusing on the type of plasmid, a similar trend was observed when comparing MC to PP, likely reflecting the lower SOX9 expression achieved with MC constructs, as previously corroborated in monolayer transfection assays. This outcome is consistent with earlier observations in our study, suggesting that suboptimal lipid-to-DNA stoichiometry [37] or an underestimation of the amount of plasmid used due to suboptimal purity may have compromised the performance of MC-based complexes [49, 50].

Next, it should be noted that SOX9 and COLX expression displayed marked donor-to-donor variability, a phenomenon well recognised in primary hMSCs, particularly those derived from femoral heads [56, 57]. Such interindividual differences may be influenced by donor age, clinical history, or intrinsic heterogeneity of the bone marrow microenvironment [58, 59]. Although the limited sample size of our study (two donors) may constrain the ability to identify more robust trends, MSCs from both donors consistently showed enhanced expression of key chondrogenic markers (SOX9, ACAN, COLII) following DP20CQ transfection. Therefore, additional studies with larger donor cohorts will contribute to further validating these findings.

Remarkably, and in good agreement with previous reports [60], overexpression of SOX9 did not affect cell densities in hMSC aggregates, as determined by H&E staining. Finally, biochemical analyses, consistent with histomorphometry findings, revealed a marked increase in proteoglycan biosynthesis in aggregates transfected with DP20CQ niosomes, whether complexed with PP or MC, compared to both negative control and LPF-transfected groups. Notably, in line with previous observations [13], LPF transfection resulted in reduced total DNA and protein content relative to aggregates transfected with DP20CQ-based formulations. Again, these findings reinforce the importance of selecting the appropriate vector to achieve the desired cell phenotype [61].

The non-viral SOX9 delivery system developed here represents an up-and-coming translational platform for focal cartilage repair. Specifically, SOX9-loaded niosomes can be readily integrated into gene-activated scaffolds [12] and implanted directly into cartilage defects enabling localized and transient gene transfer to endogenous or implanted MSCs. Such an in situ gene-modulatory environment may enhance the formation of hyaline-like repaired tissue and help prevent secondary degeneration of the surrounding cartilage, a key factor in delaying osteoarthritis progression. Moreover, this strategy could be applied either as a standalone intervention for early cartilage lesions or as an adjuvant to established reparative techniques such as microfracture or matrix-assisted cartilage repair. While further in vivo validation will be required, these findings highlight the potential of niosome-mediated SOX9 delivery as a clinically relevant platform within current cartilage regenerative paradigms.

## Conclusion

Niosomes represent a promising non-viral gene delivery system for the genetic modification of primary human bone marrow-derived mesenchymal stem cells (hMSCs). Among the tested formulations, DP20CQ niosomes composed of DOTMA (D), polysorbate 20 (P20), and chloroquine (CQ) complexed with either the parental plasmid (PP) encoding GFP and SOX9 as reporter and therapeutic gene, respectively, or its corresponding minicircle DNA (MC), exhibited suitable physicochemical properties and transfection levels that did not differ statistically from those of Lipofectamine (LPF) under selected conditions. Of note, SOX9 overexpression via DP20CQ niosomes complexed with PP plasmid enhanced chondrogenic differentiation within a 3D hMSC aggregate culture model, promoting the expression of key cartilage-specific markers while attenuating hypertrophy and osteogenic phenotypes. Further optimisation of PP and MC dose and purity may reveal superior transfection efficiency and therapeutic benefit of MC vectors.

Taken together, these findings underscore the suitability of niosome-based gene delivery systems to overcome current limitations in MSCs gene therapy and to support the development of cell-based treatments for cartilage repair. From a translational perspective, SOX9-loaded niosomes may represent a promising platform for the development of gene-activated scaffolds or for adjuvants to current cartilage repair strategies, pending in vivo validation.

## Supplementary Information

Below is the link to the electronic supplementary material.

Supplementary Material 1.

Supplementary Material 2.

Supplementary Material 3.

## Data Availability

No datasets were generated or analysed during the current study.
