# Supplementary material for: Targeted chondrogenic differentiation of human MSCs using niosomes for SOX9 gene delivery: comparison of minicircle and conventional plasmids
Source: Stem Cell Res Ther. 2025 Dec 25;17:52. doi: 10.1186/s13287-025-04867-5 (PMC12849684; doi:10.1186/s13287-025-04867-5)
Supplement: Supplementary file 1 — Supplementary Material 1. [file 13287_2025_4867_MOESM1_ESM.docx]

**Additional file 1**

**Table S1**. **Polydispersity index (PDI) of niosomes and nioplexes.** PDI values of niosome (0) and nioplexes based on polysorbate 20 and chloroquine (DP20CQ) or polysorbate 80 and cholesterol (DP80CH) and complexed with either parental plasmid (PP) or minicircle (MC) at 5/1 and 10/1 DOTMA/DNA ratios. Data are shown as mean ± SD.

| Formulation | DOTMA/DNA ratio (w/w) | Plasmid | PDI |
| --- | --- | --- | --- |
| **DP20CQ** | 0 (niosome) | | 0.400 ± 0.004 |
|  | 5/1 | PP | 0.316 ± 0.055 |
|  |  | MC | 0.466 ± 0.092 |
|  | 10/1 | PP | 0.477 ± 0.077 |
|  |  | MC | 0.333 ± 0.017 |
| **DP80CH** | 0 (niosome) | | 0.364 ± 0.003 |
|  | 5/1 | PP | 0.473 ± 0.051 |
|  |  | MC | 0.313 ± 0.051 |
|  | 10/1 | PP | 0.315 ± 0.039 |
|  |  | MC | 0.477 ± 0.024 |
