# Supplementary material for: Targeted chondrogenic differentiation of human MSCs using niosomes for SOX9 gene delivery: comparison of minicircle and conventional plasmids
Source: Stem Cell Res Ther. 2025 Dec 25;17:52. doi: 10.1186/s13287-025-04867-5 (PMC12849684; doi:10.1186/s13287-025-04867-5)
Supplement: Supplementary file 2 — Supplementary Material 2. [file 13287_2025_4867_MOESM2_ESM.docx]

**Additional file 2**


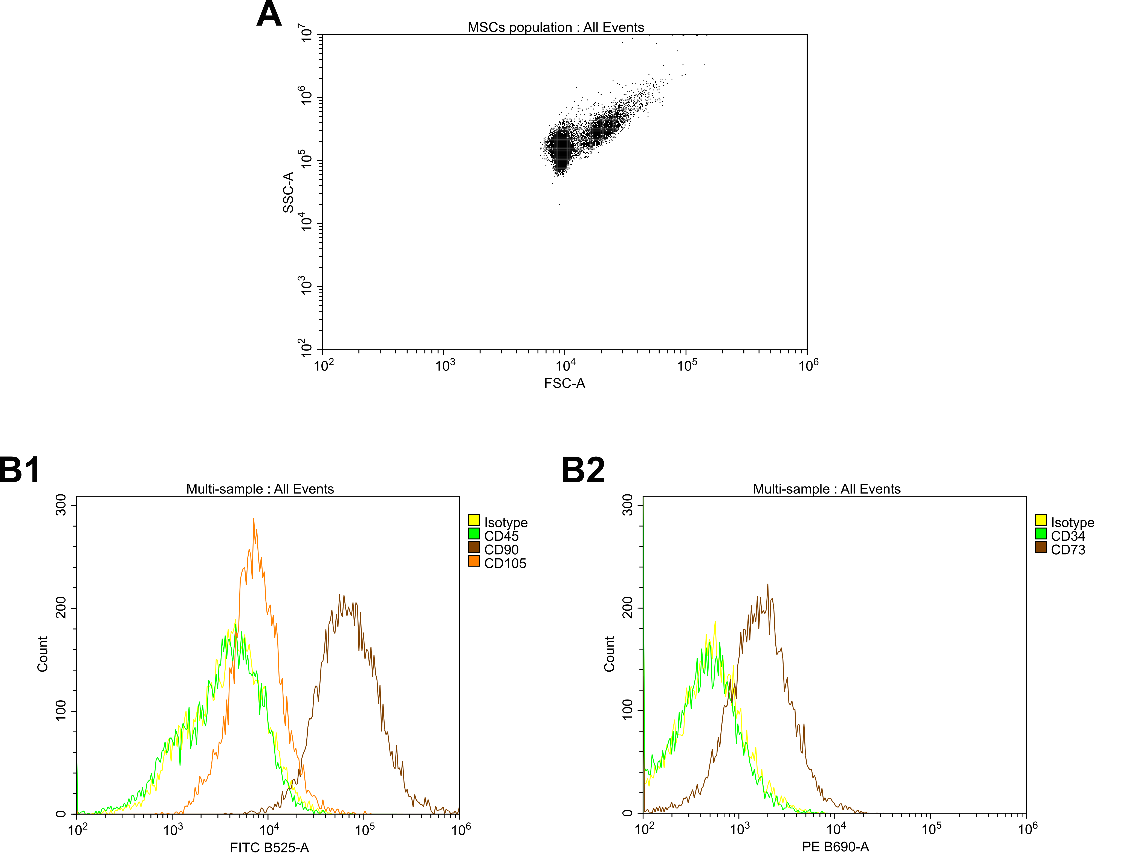


***Figure S1.* Characterization of hMSC.** Flow cytometry was used to measure surface markers CD90, CD73, and CD105, along with the hematopoietic markers CD45 and CD34, compared with their respective FITC- or PE-conjugated isotype controls as negative controls.
