# Supplementary material for: Targeted chondrogenic differentiation of human MSCs using niosomes for SOX9 gene delivery: comparison of minicircle and conventional plasmids
Source: Stem Cell Res Ther. 2025 Dec 25;17:52. doi: 10.1186/s13287-025-04867-5 (PMC12849684; doi:10.1186/s13287-025-04867-5)

Supplementary file

Gels and Blots image

Figure 1: Original full-length uncropped. Verification of both parental (PP) and minicircle (MC) plasmid by restriction enzyme treatment and agarose gel electrophoresis.

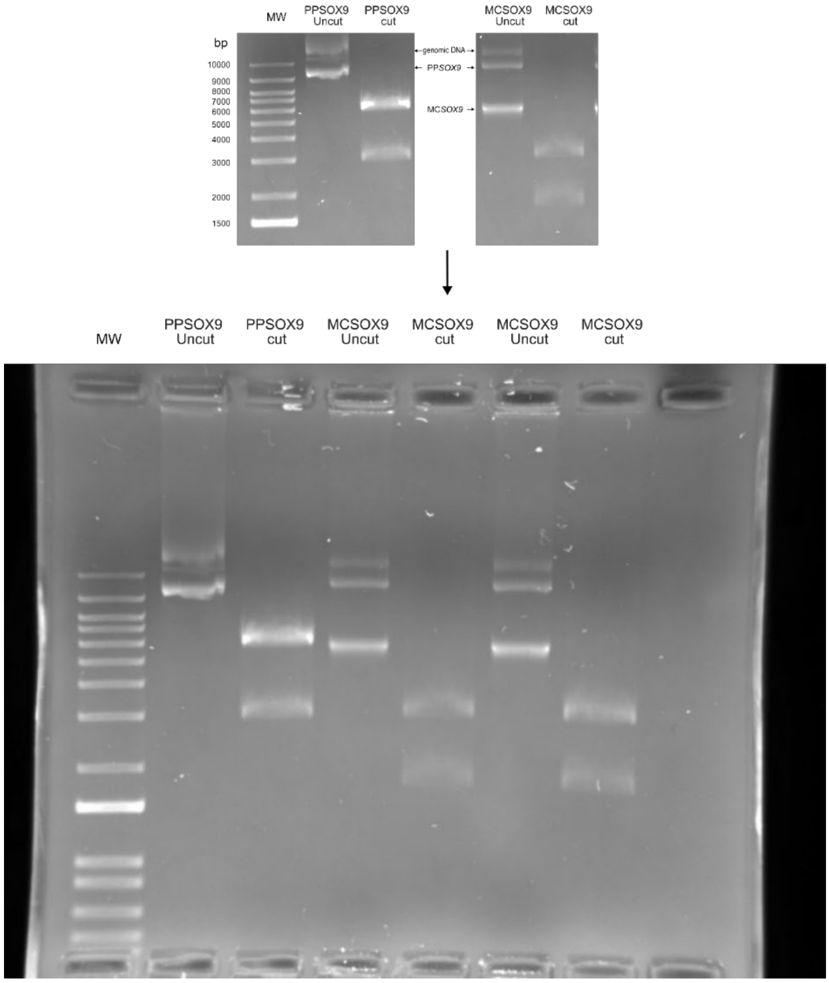

Supplement: Supplementary file 3 — Supplementary Material 3. [file 13287_2025_4867_MOESM3_ESM.pdf]
